# A study on the influence of virtual idol endorsers on consumer brand attitude

**DOI:** 10.3389/fpsyg.2026.1819749

**Published:** 2026-07-06

**Authors:** Pushen Song, Tingting Wang, Kewei Shi, Yingchun Zhao, Xiaobo Xu

**Affiliations:** 1 Sichuan Conservatory of Music, Chengdu, China; 2 Southwestern University of Finance and Economics, Chengdu, China; 3 Shanghai Normal University, Shanghai, China

**Keywords:** brand attitude, brand trust, social identity, virtual idol, virtual idol endorser

## Abstract

With the continuous development of technologies such as big data and artificial intelligence, digital idols are gradually being favored by branded enterprises. Based on meaning transfer theory and trust theory, this paper conducts four scenario experiments to explore how virtual idol endorsers influence consumers' brand attitudes, as well as the moderating effect of brand type. The study results show that virtual idol endorsers affect consumer brand attitude when they buy symbolic brand products by enhancing their social identity. When consumers buy functional brand products, virtual idol endorsers influence their brand attitude by improving their brand trust. Theoretically, this study enriches the research on the influence of virtual idol endorsers on consumer brand attitude under different brand types. Practically, it provides valuable reference for brands to formulate global virtual endorsement strategies and improve the stability and continuity of corporate brand marketing and promotion.

## Introduction

Brand endorsers are key information sources for transmitting brand messages and shaping brand image (McCracken, 1989). What started as collaborations with celebrities have extended to popular social media content creators, or social media influencers (SMIs; Bakr et al., 2026; Lou and Yuan, 2019). However, recent advances in AI technology and visual content generation have led to a new form of influencer: virtual endorsers (Bakr et al., 2026).

The emergence of virtual idol endorsers provides new marketing perspectives and creates new opportunities for brand marketing and promotion (Cheung et al., 2022; Deng et al., 2024). Virtual idol endorsers refer to fictional characters generated by computer technology, presented in virtual forms, and engaged in brand communication activities (Li et al., 2023). Compared with human endorsers, virtual idol endorsers have advantages in controllability, cost-effectiveness, and image plasticity (Song Y. et al., 2024; Zheng et al., 2024). The words and behaviors of virtual endorsers are operated by professional teams, resulting in an extremely low probability of negative events (Zheng et al., 2024). The development and maintenance costs of virtual idols are more controllable relative to human celebrities (Song X. et al., 2024). The appearance design and personality traits of virtual idols can be customized according to brand needs (Yan et al., 2024). Therefore, an increasing number of brands are using virtual idols as brand endorsers. Their application has extended from street posters in Japan to social media in China, and has penetrated the music and fashion industries in Europe and America, showing a significant trend of globalization (Zheng et al., 2024).

Consumers often pay attention to brand endorsers when choosing products. In this context, two cues are particularly important: the credibility of the endorser, which reduces consumer skepticism and enhances trust (Ohanian, 1991); and the congruence between the endorser and the brand/product, which improves the perceived authenticity and fit between them (McCormick, 2016). However, it is worth noting that the influence of these two cues is not constant; it varies depending on the nature of the endorser (e.g., virtual idol or human endorser) and the type of brand/product, thereby exerting different effects on consumers' purchase decisions. According to meaning transfer theory (McCracken, 1989), the effectiveness of endorsers stems from the cultural meanings they carry. These meanings are transferred from the endorser to the brand, then from the brand to the consumer, and finally absorbed through consumption behavior. Therefore, consumers pay careful attention to brand endorsers when selecting products. They evaluate not only the functional utility of the product but also whether the endorser can provide them with ideal self-projection and group belonging. Brand trust theory also indicates that brand trust is the degree to which consumers rely on the brand's reliability and its promises (Delgado-Ballester et al., 2003). Although some studies suggest that virtual endorsers lack human characteristics, leading to a sense of alienation and distrust toward the products they endorse (Fan et al., 2024), in reality many products still choose virtual idols as endorsers.

Existing research has shown a significant match-up effect between virtual endorsers and product types. For example, virtual idols are more suitable for recommending utilitarian products, while human endorsers are more effective in promoting hedonic products (Belanche et al., 2024). However, previous studies have often argued that the credibility of virtual idol endorsers is lower than that of human endorser (Choudhry et al., 2022; Lou, 2022). Nevertheless, these studies generally ignore that the mechanism and influence of credibility vary across different product type contexts, leading to obvious limitations in understanding the boundary conditions of virtual endorser effectiveness.

Therefore, this paper conducts four scenario experiments to examine the internal mechanism through which virtual idol endorsers influence consumers' brand attitudes, aiming to reveal the mediating roles of variables such as social identity and brand trust, as well as the moderating role of brand type. From a theoretical perspective, this research contributes to the literature by enriching the understanding of the antecedents that influence consumers' brand attitudes and advancing the study of virtual idol endorsers. From a practical perspective, it can also help companies optimize the image design and promotion strategies of virtual idol endorsers.

## Literature review

### Virtual idol endorsers and their characteristics

Since the 1990s, scholars have begun to study virtual idol endorsers. (Callcott and Lee 1994) describe virtual idol endorsers as fictional images that enterprises use to promote their products, services, or concepts. These images may be humans, animals, creatures with personality traits, or concrete product forms, used to express the brand's personality, culture, and values (Callcott and Lee, 1994). (Escalas and Bettman 2017) further define virtual idol endorsers as character images created through computer technology, embodied in virtual environments such as the Internet, who engage in performance activities. These figures do not exist in physical form but are capable of conveying a brand's personality, culture, and values to consumers. (Garretson and Burton 2005) describe them as “visual anthropomorphic representations capable of expressing brand personality, culture, and values,” which provide companies with greater flexibility, enhance brand distinctiveness and innovation reputation, and improve brand safety (Conti et al., 2022; Garretson and Burton, 2005). In essence, a virtual idol endorser refers to a fictional character created through advanced computer technology and used to promote a company's products in the online or real world. From the perspective of their typology, virtual idol endorsers include anime characters, virtual singers, virtual celebrities, and virtual streamers. These figures possess large fan bases due to their attractiveness and uniqueness. Moreover, their extensive audience reach and high levels of market attention embody substantial commercial value and marketing potential (Zhang et al., 2025). The marketing potential of virtual idol endorsers lies in their ability to transcend the boundaries of traditional marketing, establish connections with consumers in innovative ways, and create deeper emotional bonds for brands (MacInnis and Folkes, 2017).

In contrast to traditional real-person endorsers, virtual idol endorsers exhibit several distinctive advantages in disseminating brand culture and constructing brand image, including stability, security, exclusivity, malleability, cost-effectiveness, and long-term sustainability (Yang et al., 2023). Virtual endorsers that are independently developed or selected by brands generally incur relatively low development costs, making them economically advantageous. Moreover, enterprises can tailor exclusive virtual idol endorsers according to their brand personality, product style, and other brand-specific elements, reflecting a high degree of exclusivity. In addition, compared with real-person endorsers, one of the most prominent characteristics of virtual idol endorsers is their security and stability. Operated and managed by professional teams, virtual idol endorsers demonstrate more consistent performance, significantly lower risk exposure, and extended life cycles. These qualities allow brands to avoid the potential reputational damage that may result from real-person endorsers' scandals or controversies (Singh et al., 2020). In the current era of rapid digital technological advancement, virtual personas and their interactive dynamics with fans increasingly resemble real-life engagements. Consequently, virtual idol endorsers exhibit a strong degree of appeal compared to traditional celebrity endorsers (Chung and Cho, 2017). Furthermore, in their routine promotional activities, virtual idols not only endorse products but also engage in intensive and intimate interactions with fans, thereby serving as effective transmitters of brand image (De Veirman et al., 2017). As such, the application of virtual idol endorsers not only narrows the psychological distance between the brand and consumers but also brings substantial marketing returns for enterprises.

### Research on virtual idol endorsers

The current research on virtual idols mostly focuses on cognitive science, communication studies, and marketing promotion, and discusses aspects such as cognitive mechanisms, circle cultures, and marketing communication. Scholars in the field of cognitive science have found that the “surreal” attributes of virtual idols (such as perfect appearance and unlimited controllability) may trigger cognitive conflicts, and users may experience the “uncanny valley effect” (MacDorman and Chattopadhyay, 2016). In communication studies, virtual idols are regarded as a medium for forming circles under the logic of emotions and relationships (Liu, 2023; Stein et al., 2024). They are considered symbolic signs of different circles, and fans can construct identity relationships through code dissemination, creative regeneration of texts, and relationship imagination (Ramasubramanian and Kornfield, 2012). In the field of marketing, scholars argue that the human-like appearance and voice of virtual idols can enhance consumers' perception of the emotional value of products, increase their trust in the products, influence purchasing decisions, and ultimately improve brand loyalty and purchase intention (Brooks et al., 2021; Han, 2021).

At present, existing research predominantly focuses on the marketing communication advantages and communication effects of virtual idol endorsers. Virtual idol endorsers attract consumer attention through their notable appeal, popularity, and influence, thereby enhancing brand perception (Wang and Lin, 2011). By interacting within advertising scenarios and offering personalized services, they fulfill consumers' psychological needs for autonomy, belonging, and competence, which in turn strengthens consumers' identification with products or services and enhances their purchase intentions (Wei et al., 2023). The fan effect associated with virtual idol endorsers is particularly significant, as it can stimulate fans' willingness to purchase and even motivate them to actively participate in brand communication, becoming a vital force in word-of-mouth marketing (Choudhry et al., 2022). In addition, some studies focus on the communication effectiveness of virtual idol endorsers. Virtual idols, through their unique modes of interaction on social media, can build emotional bonds with fans, thereby increasing brand loyalty and user stickiness (Yap and Ismail, 2022). Enhancing the anthropomorphism and perceived realism of virtual idols has been shown to attract consumers, boost brand attachment, and improve promotional outcomes (Allal-Chérif et al., 2024). Owing to their distinctive styles, virtual idols can also provide unique and novel content to capture consumer attention and stimulate purchase desires (Sookkaew and Saephoo, 2021). However, while existing studies examine virtual idol endorsers from multiple perspectives, there remains a lack of in-depth analysis of the psychological mechanisms through which virtual idol endorsers influence consumer brand attitude. Furthermore, current research has largely overlooked the fit between virtual idols and different brand types, as well as the differentiated psychological and behavioral characteristics of consumers when purchasing various categories of products.

In summary, although existing studies have explored the functional mechanisms and marketing communication effectiveness of virtual idol endorsers, few have examined the underlying psychological mechanisms through which virtual idol endorsers influence consumer brand attitude. Moreover, there is a lack of detailed investigation into the applicability of virtual idol endorsers across different brand types and the varying degrees of influence they exert across different brand categories. Therefore, this study draws on the theories of trust and meaning transfer to explore the influence of virtual idol endorsers on consumers' brand attitudes under different brand types.

## Model construction and hypothesis development

### Virtual idol endorsers and consumer brand attitude

An information source refers to the sender of a message, who transmits information through specific channels to the intended audience (Kotler et al., 2014). Due to consumers' admiration for celebrities, products endorsed by celebrities under otherwise similar conditions tend to possess higher credibility and greater appeal (Cabeza-Ramírez et al., 2022). Studies have shown that celebrity endorsers can strengthen the positive association between consumers who already hold a favorable attitude toward a brand and the brand itself (Min et al., 2019; Thwaites et al., 2012). According to (Amos et al. 2008), when celebrities endorse a brand, consumers tend to associate the brand with the celebrity, leading to a more favorable brand attitude. (Özer et al. 2022) further demonstrated that celebrity endorsers can enhance consumers' brand attachment and brand loyalty. Moreover, previous research has consistently found that, as an information source, endorsers can positively influence consumer brand attitude. The findings of (Felix and Borges 2014) also confirmed that a well-matched endorser can lead to more favorable consumer evaluations of both the advertisement and the brand.

Although virtual idol endorsers rely on simulation technology to present anthropomorphic images, their daily interactions are largely indistinguishable from those of real human endorsers. They are often endowed with distinct personalities and character traits, and they exhibit human-like attributes in terms of language expression and behavior. In some cases, virtual idols may even appear more natural than their human counterparts (Arsenyan and Mirowska, 2021; Dabiran et al., 2024; Guthrie, 2020; Sands et al., 2022). In addition, when the virtual idol endorses, the fans participate in the process of the formation of their personality, and the interaction with the fans also deepens the high degree of adhesion between the fans and the formation of a certain private domain traffic and influence (Jin et al., 2019). Therefore, the social capital of virtual idol endorsers—including traffic, fan base size, and influence—is comparable to that of real celebrity endorsers. As products of the digital age, virtual idol endorsers are also capable of exerting a positive influence on consumer brand attitude. Accordingly, this study proposes the following hypothesis:

*H1*: Virtual idol endorsers (vs. absence) has a significant positive effect on consumer brand attitude.

### The mediating role of social identity

According to the meaning transfer theory, when the endorser transfers the identity characteristics symbolized by themselves to the brand, consumers are motivated to purchase the product in order to acquire the “meaning” embodied by the endorser. This enables them to construct their self-concept and convey identity characteristics through consumption, thereby signaling their social image and status *via* the brand (McCracken, 1986). When a brand establishes associations with specific celebrities through endorser partnerships, consumers, guided by the mechanism of symbolic meaning transfer, tend to infer brand attributes based on the endorser's recognizable traits—such as sincerity, innovativeness, or markers of social status—which are transferred to the brand through this symbolic association process (Tian et al., 2022). For symbolic brands, their core value lies in enabling consumers to express social status and fulfill needs related to leisure and entertainment (Belk, 1985; Hirschman and Holbrook, 1982). Consumers generally attach greater importance to the external symbolic attributes of such products (Sela and Berger, 2012). Therefore, when engaging with symbolic consumption, consumers tend to focus on whether the product aligns with their self-image, enhances their social standing, and facilitates the expression of their self-concept (Dhar and Wertenbroch, 2000). Virtual idol endorsers are characterized by distinct visual identities, unique personalities, and specific value orientations. They integrate popular cultural elements and symbolic motifs, endowing them with strong semiotic significance (Huang et al., 2022). When such symbolic meanings are aligned with the brand positioning of symbolic products, they significantly enhance the symbolic value of the brand. As a result, consumers are able to clearly perceive the social identity and status represented by the brand.

Social identity refers to the process by which individuals perceive themselves as belonging to a particular social group, a process that carries emotional and value-based significance (Deaux, 1993). Consumers have a psychological need for social identity and often seek to express their group affiliation through consumption behaviors in order to gain recognition and respect from others (Belk, 1985). When virtual idols endorse symbolic brands, meanings such as trendiness, fashion, confidence, and charisma associated with the virtual idol are transferred to the endorsed brand (McCracken, 1986). Consumers who purchase such branded products associate themselves with the social groups represented by the virtual idols. They believe that by using these products, they can acquire traits similar to those of the virtual idols—such as being fashionable and trendy—thereby integrating into a group that possesses a specific social identity and status. This enhances their sense of social identity. At this point, consumers perceive the brand as aligning with their ideal self-concept and aspirational social identity, resulting in favorable perceptions of the brand, increased trust, and enhanced brand loyalty. They are also more willing to pay a premium for the brand and more likely to recommend it to others, ultimately forming a positive brand attitude (Roy and Jain, 2017). Based on the above, we propose the following hypothesis:

*H2a*: For symbolic brands, the presence (vs. absence) of virtual idol endorsers increases consumers' social identity.

*H2b*: Social identity positively influences consumer brand attitude.

### The mediating role of brand trust

Brand trust refers to the willingness of consumers to expect that a brand will deliver positive outcomes, and to continue trusting it even under conditions of uncertainty or risk (Delgado-Ballester et al., 2003). Brand trust plays a critical role in shaping consumer decision-making behavior, as it strengthens the connection and identification between consumers and brands. This emotional bond effectively reduces consumers' perceived risk and significantly enhances their purchase intention (Shamim et al., 2024). For functional brands, consumers are more concerned with product performance, quality, practicality, and reliability (Hirschman and Holbrook, 1982; Strahilevitz and Myers, 1998). Therefore, the credibility and reliability of the endorser are often more likely to attract consumer attention and trust (Kong and Fang, 2024). Compared with real celebrity endorsers who may be involved in controversies, virtual idol endorsers are highly controllable and stable, which enables them to maintain a consistently positive public image. This allows brands to ensure that the endorsers communicate their core values and advantages effectively (Bu et al., 2022; Lou et al., 2023; Ozdemir et al., 2023). Moreover, the credibility of virtual idol endorsers can significantly enhance consumers' trust in the endorsed brand (Garretson and Burton, 2005). The human-like characteristics of virtual idols improve the transmission and persuasiveness of brand messages (Thomas and Fowler, 2021). During interactions with consumers, the credibility of virtual idol endorsers is further reinforced. With the help of digital technology and social media platforms, virtual idols can engage in real-time and high-frequency interactions with consumers, which significantly strengthens consumers' trust (Jin et al., 2021). This trust, developed through interaction, is naturally transferred to the brand they represent. Based on the above, we propose that for functional brands, virtual idol endorsers can effectively enhance consumers' brand trust. Furthermore, (Garretson and Niedrich 2004) empirically demonstrated that the professionalism, credibility, and reliability of virtual endorsers jointly contribute to brand trust, which in turn leads to sustained purchasing behavior and a favorable brand attitude.

Therefore, the following hypotheses are proposed:

*H3a*: For functional brands, the presence (vs. absence) of virtual idol endorsers increases consumers' brand trust.

*H3b*: Brand trust positively influences consumer brand attitude.

**Figure 1:**
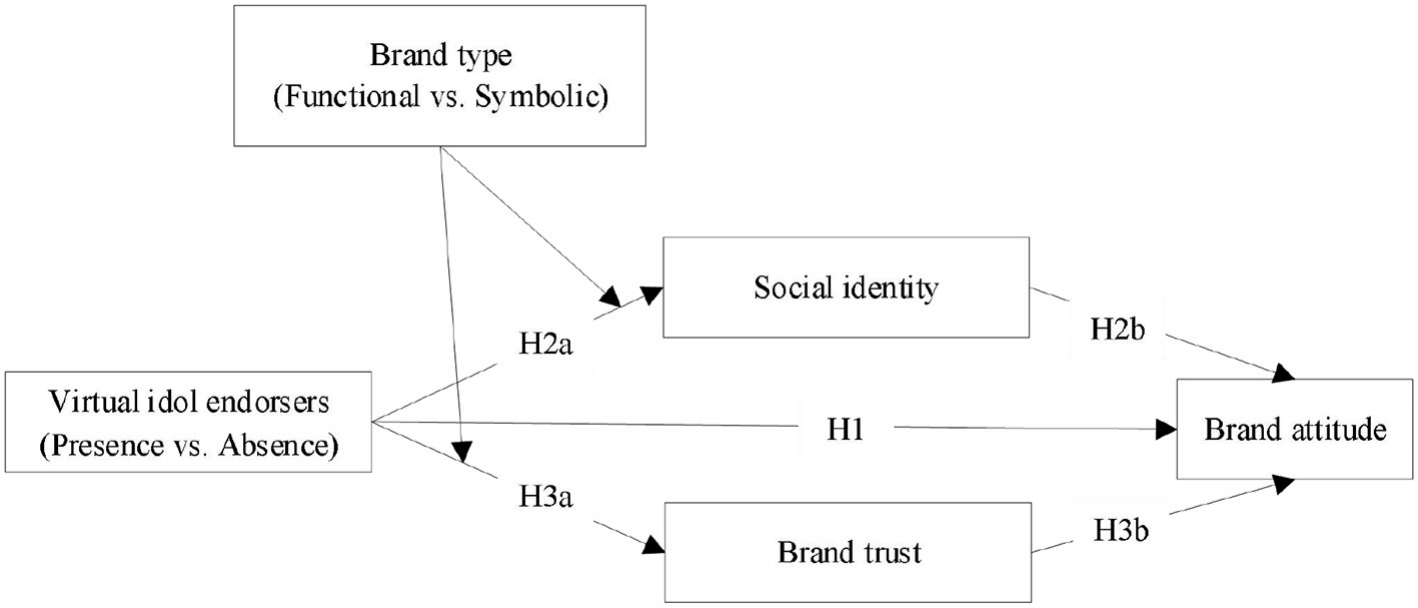
The research model.

In summary, the theoretical model is constructed as shown in Figure 1. In this paper, four scenario experiments are used to test the above hypothesis. Among them, Study 1a and Study 1b verified the influence of the virtual idol endorsers (vs. absence) on consumer brand attitude. Based on different brand backgrounds, Study 2a and Study 2b analyzed the mediating role of social identity and brand trust based on Study 1 and introduced the moderating variable of brand type to explore further the effects of different brand types (functional vs. symbolic) the difference of influence of virtual idol endorsers on consumer brand attitude. The detailed study design is presented in Table 1.

**Table 1:** Study design.

Study	Experiment design	Hypothesis	Scenario
Study 1a Study 1b	Virtual endorsers: presence vs. absence	H1	In the context of the same brand, virtual endorsers: presence vs. absence
Study 2a Study 2b	2 (Virtual endorsers: presence vs. absence) × 2 (Brand types: functional vs. symbolic)	H2a, H2b, H3a, H3b	Under different brand backgrounds, functional brands vs. symbolic brands

## Study 1

Study 1 conducts a preliminary verification of the main effect of the full test, namely the influence of virtual idol endorsers on consumer brand attitude. The participants in Study 1 were Chinese subjects. By comparing these two sets of data, this test aims to explore whether virtual idols have a positive impact on consumers' attitudes toward brands.

### Design and participants

Study 1 employed a one-factor between-subjects experimental design (virtual idol endorser: presence vs. absence) to examine the impact of virtual idol endorsers on consumer brand attitude and their cross-cultural applicability. Two well-known virtual idols—Hatsune Miku and AYAYI—were selected as endorsers for Study 1a and Study 1b, respectively. Hatsune Miku, a globally recognized virtual idol, has attracted millions of fans through her iconic appearance and holographic concerts. AYAYI, on the other hand, is China's first hyper-realistic digital human, who gained rapid popularity on Chinese social media due to her ultra-realistic appearance and has endorsed several internationally renowned brands.

**Figure 2:**
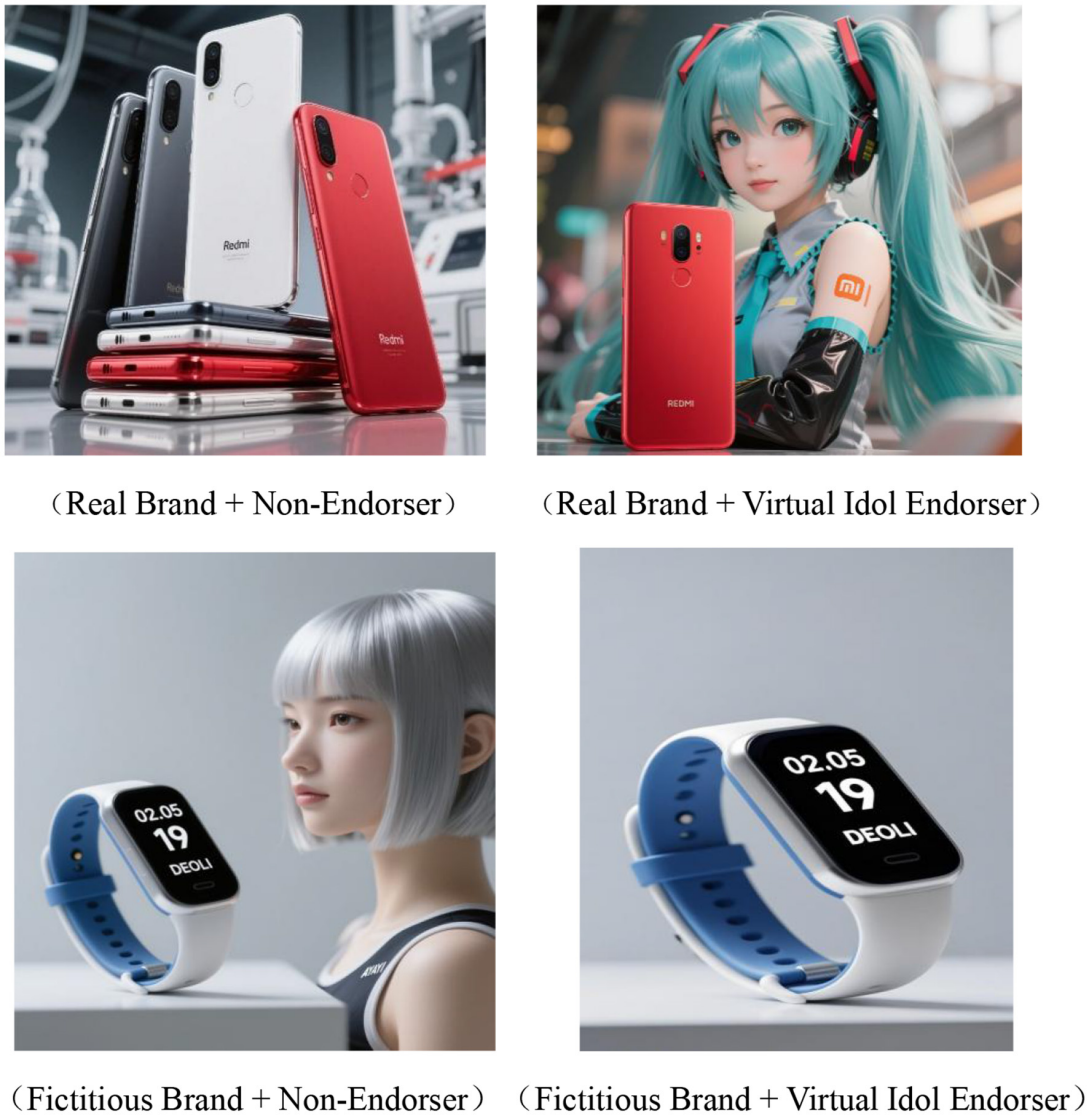
The experimental materials for experiment 1. Source: Made by the author using Doubao AI website.

In Study 1a, a real smartphone brand—Xiaomi—was used, with Hatsune Miku serving as the virtual endorser (Figure 2). To eliminate potential confounding factors such as brand familiarity and brand type, Study 1b adopted a fictitious sports wearable brand—“FitCore”—and selected AYAYI as the virtual endorser (Figure 2).

Both studies were conducted online *via* Sojump.com. A total of 107 Chinese participants took part in Study 1a (40.2% Female, Mage = 24.11, SD = 1.554).

## Study 1a

### Procedure and measurement

Participants were first instructed to read a product introduction and view the corresponding marketing promotional image. Following this, they were asked to report their brand attitude. The measurement items for brand attitude were adapted from (Ohanian 1990) validated scale, comprising six items (Study 1a: α = 0.956; Study 1b: α = 0.820), such as: “I think this brand is professional,” “I think this brand is of high quality,” “I think this brand is trustworthy.” Responses were recorded on a 7-point Likert scale (1 = strongly disagree, 7 = strongly agree). Finally, demographic information was collected, including participants' gender and age. See Appendix 1 for the full list of measurement items and results of the confirmatory factor analysis.

### Results

ANOVA results indicated that in Study 1a, brand attitude was significantly higher in the virtual idol endorser condition than in the no-endorser condition [MAbsence = 4.01, SD = 1.787; MPresence = 5.19, SD = 1.265; *F* (1,105) = 15.321, *p* < 0.001, η2 = 0.127]. Thus, H1 was supported.

## Study 1b

### Study design

Study 1b is a field experiment conducted on the Douyin platform to examine the impact of virtual idol endorsers (presence vs. absence) on consumers' brand attitudes (H1).

The experiment selected China's first hyper-realistic digital human, AYAYI, as the endorser, and used the fictional sports wearable brand “FitCore” as the test object. Two sets of videos were set up: one with a virtual idol endorser (AYAYI appeared) and the other without an endorser (only the product was displayed). The experimental group not only introduced the products of “FitCore” brand but also presented the virtual idol AYAYI as the brand's endorser; the control group viewed the same product introduction videos without any endorsers, to rule out irrelevant interfering factors.

### Study process

The formal experiment was carried out on the Douyin platform. First, to avoid the interference of the account's past experience and fans on the video marketing results, the researchers established a new account on Douyin. Then, two experimental videos were simultaneously posted on Douyin, with the time and title of the videos being consistent, but the video content being different. Later, the researchers purchased 1,500 exposures for each video through the official promotion channels of Douyin and set “not to send multiple videos to the same viewer” and “prevent accessing the homepage” to prevent the subjects from being influenced by watching multiple videos. After 24 h, the number of views and likes of each video was counted (Table 2). Users' like behaviors can be regarded as positive brand attitudes (Sun and Lv, 2025). Therefore, we used the like rate as the measurement method for brand attitudes.

**Table 2:** The effect of advertising video placement.

Endorser types	Views	Likes	Likes/views
With virtual idol endorser	1,571	49	3.12%
Without virtual idol endorser	1,683	33	1.96%

### Results

The chi-square test results showed that compared to the group without virtual idol endorsers, the advertising effect of the group with virtual idol endorsers was significantly better [χ^2^(1) = 4.437, *p* = 0.035 < 0.050], indicating that consumers were more inclined toward products with virtual idol endorsements. Therefore, H1 was supported.

Study 1b used a field experiment to examine the influence of virtual idol endorser (presence vs. absence) on consumers' brand attitudes. The research results proved that virtual idol endorsers had a positive effect on consumers' brand attitudes.

Compared with Study 1a, which used a real brand (Xiaomi mobile phones) and a Japanese virtual idol, Hatsune Miku, and conducted the research in the form of an online questionnaire, Study 1b replaced the fictional brand with a Chinese local hyper-realistic virtual idol, AYAYI, and transferred the context to a real short-video social platform, thereby eliminating the influence of brand prior knowledge, differences in virtual idol cultural background, and possible interference from the questionnaire context, significantly enhancing the robustness and external validity of the research conclusion.

### Discussion

The analysis results of Study 1a and Study 1b demonstrate the contextual generalizability of the positive impact of virtual idol endorsers on consumer brand attitude. Regardless of whether the brand is real or fictitious, the involvement of a virtual idol significantly enhances consumers' brand evaluations. Accordingly, Study 2a and Study 2b further explore the underlying mechanisms—specifically, the mediating pathways of social identity and brand trust, and the moderating boundary of brand type—to deepen the understanding of how virtual idol endorsers influence consumer brand attitude.

## Study 2a

To further explore the influence of virtual idol endorsers on consumer brand attitude, we designed Study 2a (virtual brands) to repeatedly test the conclusions of the main effect. On this basis, the mediating mechanisms of social identity and brand trust, as well as the moderating role of brand type, were also discussed and analyzed.

### Design and participants

**Figure 3:**
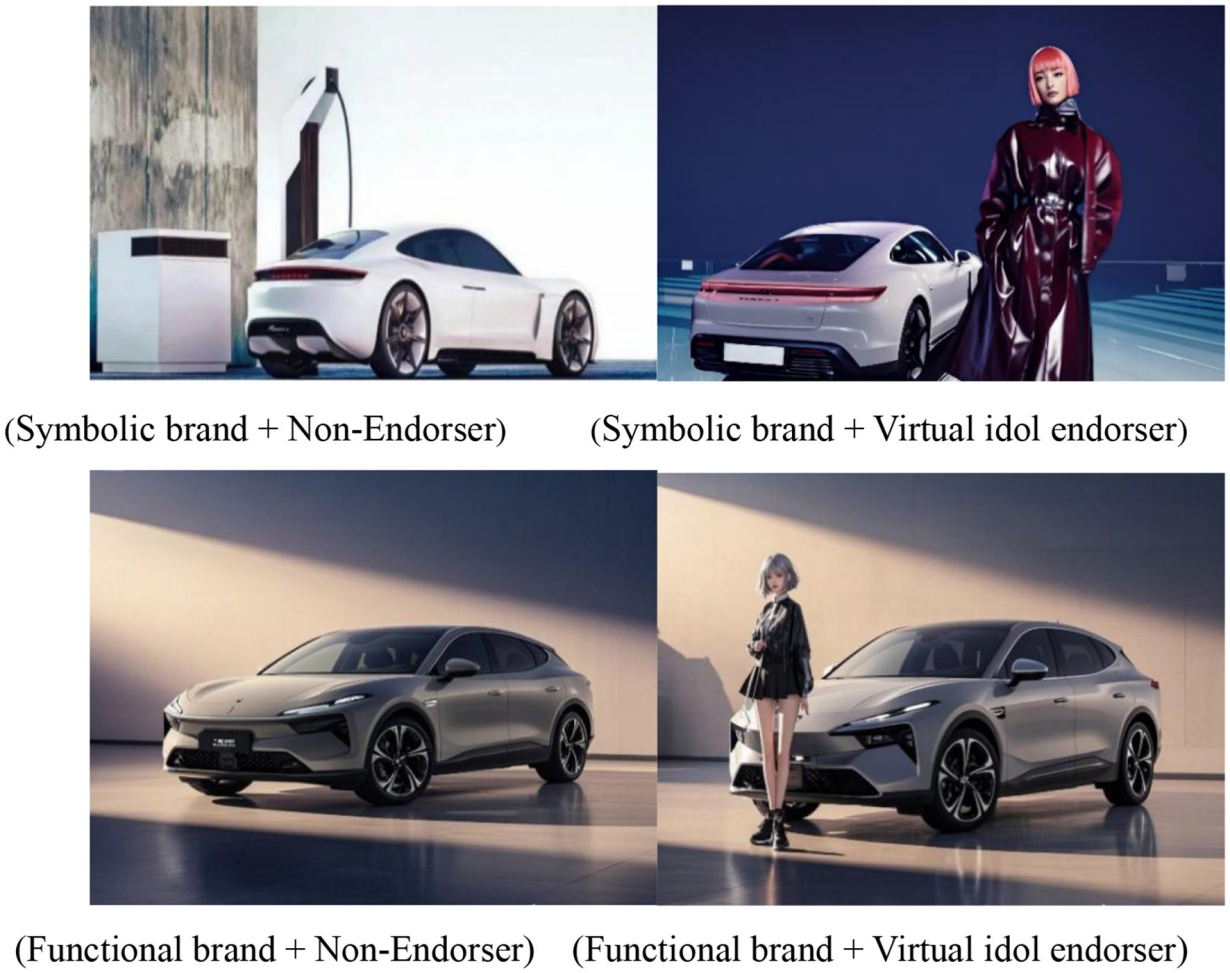
The experimental materials for Study 2a. Source: Made by the author using Doubao AI website.

**Figure 4:**
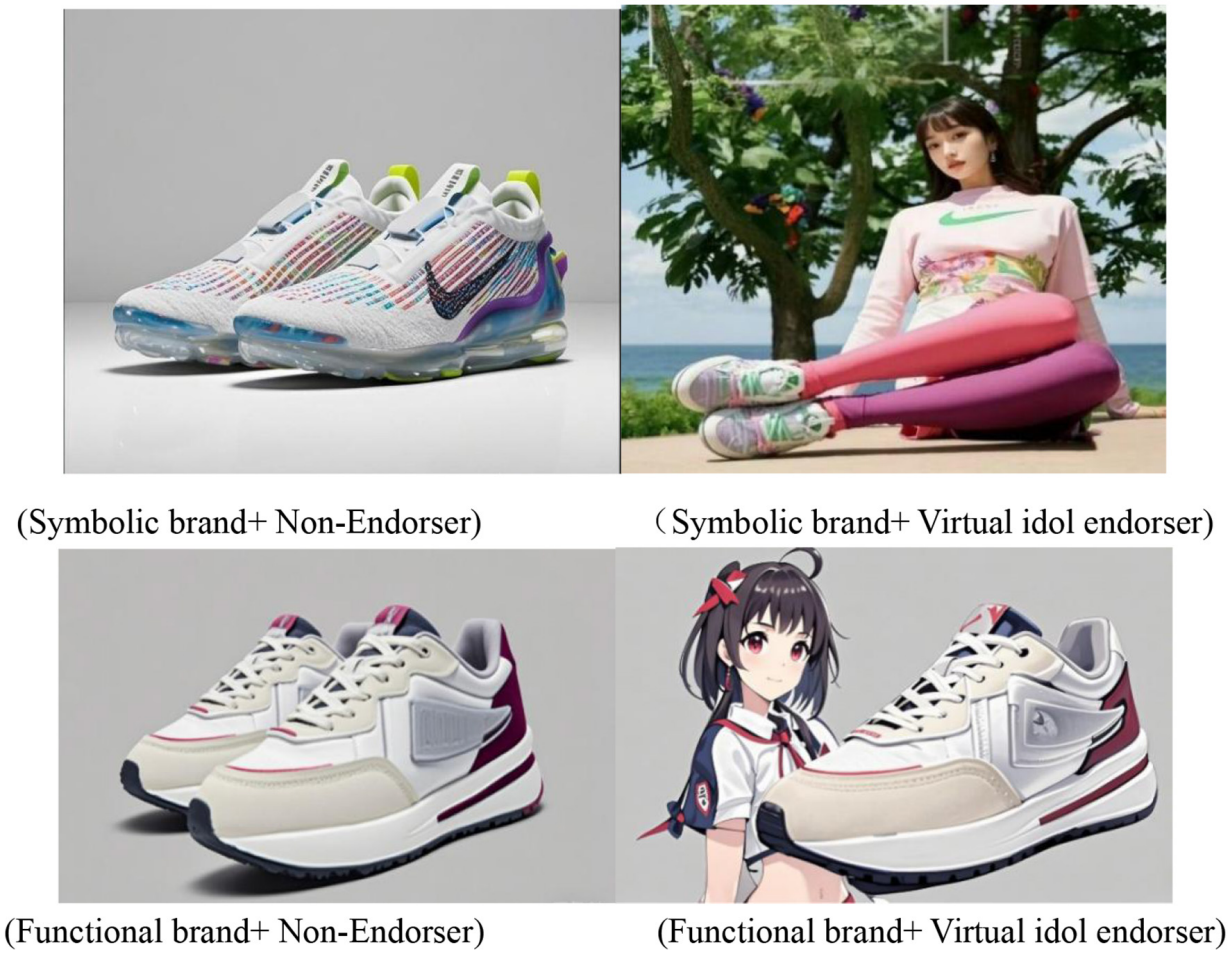
The experimental materials for Study 2b. Source: Made by the author using Doubao AI website.

Study 2a employed a 2 (automobile brand types: functional vs. symbolic) × 2 (virtual idol endorsers: presence vs. absence) between-subjects experimental design. Two fictitious automobile brands were created to eliminate the potential influence of participants' pre-existing perceptions of real-world brands. Regarding the selection of virtual idols, Study 2a chose two well-known virtual figures: IMMA and Gong Chengshi. IMMA is a hyper-realistic Japanese virtual fashion icon who has graced magazine covers, collaborated with commercial brands, and gained significant popularity on social media. Gong Chengshi, on the other hand, is the first virtual human launched by China's automobile industry, designed to help brands connect with younger audiences through innovative campaigns such as virtual product launches and cross-dimensional interactions. Specifically, IMMA (see Figure 3) was selected as the virtual endorser for the symbolic brand “Pamela Auto”, while Gong Chengshi (see Figure 4) endorsed the functional brand “Ping An Auto”. Study 2a was conducted on the professional Chinese survey platform WJS, a leading tool for online research and data collection. A total of 180 Chinese participants took part in Study 2a (54.40% Female; Mage = 31.22, SD = 7.732).

### Pretest

This study conducted a pretest to validate the manipulation of brand types. Following the study of (Sweeney and Soutar 2001), we developed two brand descriptions: one for a functional brand—Ping An Auto, and the other for a symbolic brand—Pamela Auto. The functional brand description emphasized product functionality, practical use, and utilitarian value, while the symbolic brand highlighted emotional benefits, cultural meaning, and social symbolism. A total of 110 Chinese participants (47.30% Female; Mage = 32.15, SD = 7.158). They were involved this pretest and were randomly assigned to read one of the two brand descriptions. Afterward, they were asked to rate their perceptions of the brand image. For the symbolic brand, perception was measured using five items adapted from (Sweeney and Soutar 2001) and (Wallace et al. 2014) (α = 0.816): “The products of this brand match my personality”; “I think that this product is used by people like me”; “The products of this brand fit my social role”; The measurement items of the functional brand were adapted from (Sweeney and Soutar 2001) validated scale, comprising 5 items (α = 0.893): “This product has many functions and I'm very satisfied”; “After using the products of this brand, I am satisfied with its performance”; “The overall quality of the products of this brand is very good” (1 = very disagree, 7 =very agree). See Appendix 1 for the full list of items and result of confirmatory factor analysis.

The results of the one-way ANOVA analysis revealed that the symbolic brand manipulation material scored significantly higher than the functional brand on the symbolic dimension [Msymbolic = 5.88, SD = 0.570; Mfunctional = 4.62, SD = 1.014; *F*(1,108) = 64.280, *p* < 0.001, η2 = 0.373]. Conversely, the functional brand manipulation material exhibited significantly higher scores than the symbolic brand on the functional dimension [Msymbolic = 4.35, SD = 0.646; Mfunctional = 5.51, SD = 0.871; *F*(1,108) = 62.539, *p* < 0.001, η2 = 0.367]. These findings indicate that the manipulation of brand types in Study 2a was successfully established.

### Procedure and measurement

First, the participants were invited to read the product introduction information and promotional posters (see Figure 4) of the virtual car brands—Ping an Auto and Pamela Auto. Then, the participants were asked to fill in the questionnaire based on their feelings after reading.

The questionnaire consists of three parts. The first part is the measurement of consumer brand attitude, social identity and brand trust. The second part is the measurement of brand type; The third part is the measurement of demographic information.

The measurement items of brand attitude were adapted from Ohanian's (1990) validated scale (α = 0.860), the same as Study 1.

Those of social identity were adapted from the validated scale of (Cheek et al. 1994) and (Johnson et al. 2004), comprising 6 items (α = 0.862): “I think the concept conveyed in this brand is the mainstream concept of society”; “Buying the products of this brand makes me feel that I have become one of the vast majority”; “The values conveyed by this brand make me feel a strong sense of belonging”.

Those of brand trust were adapted from the validated scale of (Dhar and Wertenbroch 2000), comprising (α = 0.872): “I think this brand meets my expectations”; “I have great confidence in this brand”; “I think this brand has never let me down”.

Those of symbolic brand were adapted from the validated scale of (Sweeney and Soutar 2001) and (Wallace et al. 2014), comprising 5 items (α = 0.924), the same as the pretest.

Those of functional brand were adapted from the validated scale of (Sweeney and Soutar 2001), comprising 5 items (α = 0.823), the same as the pretest. All the items adopted a 7-point scale (1 = very disagree, 7 = very agree). See Appendix 1 for the full list of items and result of confirmatory factor analysis.

### Results

Manipulation check. To examine the effectiveness of the brand type manipulation, a one-way ANOVA analysis was conducted, and the results confirmed its success: symbolic brands scored significantly higher than functional brands on the symbolic dimension [Msymbolic = 5.64, SD = 0.616; Mfunctional = 4.64, SD = 0.907; *F*(1,178) = 76.148, *p* < 0.001, η2 = 0.300]; functional brands scored significantly higher than symbolic brands on the functional dimension [Msymbolic = 4.63, SD = 0.785; Mfunctional = 5.64, SD = 0.640; *F*(1,178) = 89.627, *p* < 0.001, η2 = 0.335].

Dependent measures. The virtual endorser group consistently exhibited higher brand attitude scores compared to the no-endorser group in either case of the symbolic brand [MPresence = 6.16, SD = 0.526, MAbsence = 5.00, SD = 0.395, *t* = −11.789, *p* < 0.001, η2 = 0.612] or the functional brand [MPresence = 6.14, SD = 0.444, MAbsence = 5.21, SD = 0.392, *t* = −10.490, *p* < 0.001, η2 = 0.587). H1 was supported again.

Moderate mediation effect. The independent variable “virtual idol endorser” and the moderating variable “brand type” were converted into dummy variables, with “no virtual idol endorser” coded as “0” and “virtual idol endorser” coded as “1”. Functional brands were assigned “2”, and symbolic brands were assigned “3”. Using virtual idol endorsers (presence vs. absence) as the independent variable, brand types (functional vs. symbolic) as the moderating variable, brand trust and social identity as mediating variables, and brand attitude as the dependent variable, the analysis was conducted *via* the bootstrapping method (PROCESS, Model 8, with 5,000 samples and 95% confidence intervals).

The results indicated that, for symbolic brands, social identity partially mediated the impact of virtual idol endorsers (presence vs. absence) on brand attitude [indirect effect = 0.075, SE = 0.033, 95% CI = (0.018, 0.146), not included 0], while brand trust failed to demonstrate a mediating effect [indirect effect = −0.032, SE = 0.028, 95%CI = (−0.099, 0.007), included 0]. Notably, the direct effect of virtual idol endorsers on brand attitude remained statistically significant [direct effect = 1.062, SE = 0.096, 95%CI = (0.873, 1.251), not included 0]. H2a and H2b was supported. H2a and H2b were supported.

For functional brands, brand trust partially mediated the effect of virtual idol endorsers (presence vs. absence) on brand attitude [indirect effect = 0.146, SE = 0.070, 95%CI = (0.015, 0.293), not included 0], while social identity did not demonstrate a mediating effect [indirect effect = 0.034, SE = 0.025, 95%CI = (−0.015, 0.086), included 0]. The direct effect remained statistically significant [direct effect = 0.732, SE = 0.110, 95%CI = (0.515, 0.949), not included 0]. H3a and H3b were supported (see Table 3).

**Table 3:** The results of the analysis of moderated mediation.

Moderator	Effect	Effect	SE	95% CI
Symbolic	Direct effect	1.062	0.096	[0.873, 1.251]
	Indirect effect			
	Social identity	0.075	0.033	[0.018, 0.146]
	Brand trust	−0.032	0.028	[−0.099, 0.007]
Functional	Direct effect	0.732	0.110	[0.515, 0.949]
	Indirect effect			
	Social identity	0.034	0.025	[−0.015, 0.086]
	Brand trust	0.146	0.070	[0.015, 0.293]

### Discussion

Building on the main effect tests in Study 1, Study 2a further validated the mediating effects of social identity and brand trust, as well as the moderating role of brand type. Specifically, for symbolic products, social identity partially mediated the relationship between virtual idol endorsers and consumer brand attitude; for functional products, brand trust played a partial mediating role. However, the conclusions of Study 2a were derived from a virtual brand context, leaving uncertain whether the underlying mechanism of virtual idol endorsers' impact on brand attitude would operate identically in real-world brand scenarios. To ensure the integrity of the experimental design and enhance external validity, Study 2b introduced real-world brands as a control group to revalidate the robustness of the hypothesized mechanisms.

## Study 2b

To make the study results scientific and reliable, Study 2b based on the successful main effect test of the real brand in Study 1, also adopted the scenario experiment method to re-verify the mediating effect of social identity recognition and brand trust, as well as the moderating effect of brand type on the influence of virtual idol endorsers on consumer brand attitude.

### Design and participants

Study 2b employed a 2 (sneaker types: functional vs. symbolic) × 2 (virtual idol endorsers: present vs. absent) between-subjects experimental design. In Study 2b, Ruby 9100M and Lingyuan were selected as virtual idol endorsers. Ruby 9100M, a Chinese cyborg-like virtual idol, has collaborated with international brands such as Nike, Dior, and LV to launch NFT clothing and co-branded sneakers, advocating for sustainable fashion through digital design. Lingyuan, one of China's most popular homegrown virtual idols, gained widespread attention among Generation Z for her ancient-style female character and original Chinese music blending opera elements, representing an innovative fusion of traditional Chinese art and virtual idol culture. Notably, Lingyuan previously served as an endorser for Xtep. In this study specifically, Ruby 9100M (Figure 4) was assigned as the virtual idol endorser for Nike, while Lingyuan (Figure 4) represented Xtep. Study 2b was conducted on WSJ platform, involving 180 Chinese participants (55.60% Female; Mage = 31.75, SD = 7.344).

### Pretest

A pretest was conducted to evaluate the manipulation materials related to brand types. Drawing on the methodology of (Sweeney and Soutar 2001), we developed descriptions for two brands: Xtep, representing a functional brand, and Nike, serving as an example of a symbolic brand. The functional brand description emphasized the brand's functional attributes, practical uses and usage value. In contrast, the symbolic brand highlighted emotional significance, cultural connotation, and social identity aspects.

A total of 134 Chinese participants (48.40% Female; Mage = 30.87, SD = 8.785) took part in this pretest. They randomly read one of the two brand introductions and then rated their perception of the brand image. The scales for symbolic brands (α = 0.870) and functional brands (α = 0.709) referred to the studies of (Sweeney and Soutar 2001) and (Wallace et al. 2014), the same as Study 2a (7-point scale, 1 = very disagree, 7 = very agree).

The results of the single-factor ANOVA analysis showed that the score of symbolic brand manipulation materials in the symbolic dimension was significantly higher than that of functional brands [Msymbolic = 5.12, SD = 0.666; Mfunctional = 4.20, SD = 0.528; *F*(1,132) = 78.912, *p* < 0.001, η2 = 0.374]; the score of functional brand manipulation materials in the functional dimension was significantly higher than that of symbolic brands [Msymbolic = 4.52, SD = 0.604; Mfunctional = 5.36, SD = 0.429; *F*(1,132) = 88.364, *p* < 0.001, η2 = 0.401], indicating that the manipulation of brand types in Study 2b was successful.

### Procedure and measurement

First, the participants were invited to read the product introduction information and promotional posters (see Figure 4) of real sports shoe brands—Xtep and Nike. Then, the participants were asked to fill out the questionnaire based on their feelings after reading.

The questionnaire consists of three parts. The first part is the measurement of consumer brand attitude (α = 0.860), social identity (α = 0.870) and brand trust (α = 0.959); the second part is the measurement of brand types, symbolic brand (α = 0.900) and functional brand (α = 0.745); the third part is the measurement of demographic information. All the measurement items were the same as those used in Study 2a and adopted 7-point scale (1 = very disagree, 7 = very agree). See Appendix 1 for the full list of items and result of confirmatory factor analysis.

### Results

Manipulation check. To examine the effectiveness of the brand type manipulation, the results of the one-way ANOVA analysis confirmed its success: symbolic brands scored significantly higher than functional brands on the symbolic dimension [Msymbolic = 5.53, SD = 0.639; Mfunctional = 4.18, SD = 0.631; *F*(1,178) = 203.703, *p* < 0.001, η2 = 0.534]; functional brands scored significantly higher than symbolic brands on the functional dimension [Msymbolic = 4.39, SD = 0.434; Mfunctional = 5.31, SD = 0.537; *F*(1,178) = 158.025, *p* < 0.001, η2 = 0.470].

Dependent measures. In either case of the symbolic brand (MPresence = 6.17, SD = 0.523, MAbsence = 5.20, SD = 0.338, *t* = −10.450, *p* < 0.001, η2 = 0.554) or the functional brand (MPresence = 6.07, SD = 0.468, MAbsence = 5.05, SD = 0.507, *t* = −9.938, *p* < 0.001, η2 = 0.529), the virtual endorser group consistently exhibited higher brand attitude scores compared to the no-endorser group. H1 was supported again.

Moderate mediation effect. Consistent with Study 2a, Study 2b used Bootstrapping method for the analysis (PROCESS, Model 8, with 5,000 samples and 95% confidence intervals). The results indicated that, for symbolic brands, social identity partially mediated the impact of virtual idol endorsers (presence vs. absence) on brand attitude [indirect effect = 0.153, SE = 0.051, 95%CI = (0.057, 0.256), not included 0], while brand trust failed to demonstrate a mediating effect [indirect effect = 0.052, SE = 0.038, 95%CI = (−0.001, 0.142), included 0]. The direct effect remained statistically significant [direct effect = 0.729, SE = 0.110, 95%CI = (0.513, 0.945), not included 0]. H2a and H2b were supported.

For functional brands, brand trust partially mediated the impact of virtual idol endorsers (presence vs. absence) on brand attitude [indirect effect = 0.066, SE = 0.036, 95%CI = (0.002, 0.142), not included 0], while social identity failed to demonstrate a mediating effect [indirect effect = 0.043, SE = 0.029, 95%CI = (−0.007, 0.104), included 0]. The direct effect remained statistically significant [direct effect = 0.905, SE = 0.104, 95%CI = (0.699, 1.110), not included 0]. H3a and H3b were supported (see Table 4).

**Table 4:** The results of the analysis of moderated mediation.

Moderator	Effect	Effect	SE	95% CI
Symbolic	Direct effect	0.729	0.110	[0.513, 0.945]
	Indirect effect			
	Social identity	0.153	0.051	[0.057, 0.256]
	Brand trust	0.052	0.038	[−0.001, 0.142]
Functional	Direct effect	0.905	0.104	[0.699, 1.110]
	Indirect effect			
	Social identity	0.043	0.029	[−0.007, 0.104]
	Brand trust	0.066	0.036	[0.002, 0.142]

### Discussion

The findings of Study 2b once again demonstrate that, compared to scenarios without virtual endorsers, consumers exhibit a more favorable brand attitude toward products endorsed by virtual idols. This conclusion further corroborates the positive role of virtual idol endorsers in brand endorsement. Moreover, building upon the foundation established in Study 2a and Study 1, Study 2b further validates the applicability of the underlying mechanism of virtual idol endorsers in real-world brand contexts.

## Discussion and conclusions

### Conclusions

Based on meaning transfer theory and trust theory, this paper conducts an empirical study on the influence of virtual idol endorsers on consumer brand attitude, their relationship with brand type, and the cross-cultural applicability of virtual idol endorsers. Firstly, virtual idol endorsers can significantly enhance consumer brand attitude. This paper demonstrates through Study 1a and Study 1b that in a cross-cultural context, whether for virtual or real brands, the group with virtual idol endorsers has a significantly higher brand attitude than the group without them. This conclusion aligns with the research finding that

“virtual endorsers can generate a positive brand attitude and consumer purchase behaviors.” (Zheng et al., 2024)

In the context of globalization, brand marketing faces the challenge of cross-cultural communication. Consumers' acceptance and responses to virtual idols may vary across different cultural backgrounds. However, based on the results of this study, it is confirmed that virtual idol endorsers can have a positive impact on consumers in different cultural backgrounds, indicating that virtual idol endorsers have certain cross-cultural applicability and providing new approaches and strategies for brand promotion in the international market.

Secondly, this study validated brand type's moderating effect on the influence of virtual idol endorsers on consumer brand attitude. The analysis results of Study 2a and Study 2b suggest, in the context of symbolic brands, social identity significantly enhances the influence of virtual idol endorsers on consumer brand attitude. As the main body of information transmission in symbolic brands, virtual idol endorsers shape the brand image by transmitting their own “meaning”, further promoting the establishment of the social identity and status that the brand symbolizes. Meanwhile, consumers can also highlight their identity and status, build a sense of self-identity, and express their unique selves by purchasing symbolic brands.

Additionally, the test results of Study 2a and Study 2b verify that, in the context of functional brands, brand trust significantly enhances the influence of virtual idol endorsers on consumer brand attitude. For functional brands, consumers pay more attention to the practical value such as product performance. Virtual idols, relying on their stability and reliability, can gain consumers' trust and, through quasi-social interaction, strengthen consumers' emotional connection. This not only increases consumers' familiarity and trust in the brand but also significantly improves their positive attitude toward the brand. By using virtual idol endorsers as a medium, brands continuously convey information about their corporate image to consumers and form an interactive relationship with consumers by personifying their brands. As a result, consumers' trust in the brands endorsed by virtual idols is significantly enhanced, and they are more inclined to choose these brands, thereby subtly influencing consumer brand attitude. This research finding responds to the call made by Thomas and Fowler for a deeper exploration of the impact of virtual endorsers on different product categories (Thomas and Fowler, 2021).

### Theoretical implications

First, current research on brand endorsers mainly focuses on comparisons between different types of endorsers, such as celebrities, experts, and social media influencers (Lefebvre and Cowart, 2022). These endorsers fall within the category of human endorsers. Although some studies have paid attention to virtual endorsers, they are primarily qualitative in nature (Yu et al., 2023). Second, this study responds to the call by Yu et al.:

“As the application of virtual idol endorsement becomes more common in marketing, empirical evidence on how it affects consumer purchase and product choices remains scarce, and the understanding of how virtual idol marketing operates is still very limited.” (Yu et al., 2025)

Therefore, this study adopts a quantitative research method to analyze and compare the mechanisms of virtual idol endorsers in brand marketing, and to explore their internal influence paths on consumer brand attitude, as well as the differential effects across different brand types and consumer groups. Consequently, this study not only expands the research scope of brand endorsers and virtual idol endorsers but also provides more precise and targeted theoretical guidance for brand marketing practice.

Second, at the theoretical framework level, this study enriches the application of meaning transfer theory and trust theory. Meaning transfer theory has traditionally been used to explain the transfer of symbolic meanings from human endorsers to brands (Schimmelpfennig and Hunt, 2020). This study extends it to the context of virtual idols, verifying the mechanism through which the symbolic value carried by virtual idols contributes to consumers' social identity construction. Classic brand trust theory is typically based on consumers' perceptions of a brand's reliability, honesty, and benevolence, and the role of an endorser is to enhance this trust through factors such as credibility, attractiveness, and trustworthiness. However, when the endorser itself is a virtual image without genuine subjectivity, the formation mechanism of brand trust differs. (Zhang and He 2025) found that virtual endorsers lead to a significant decrease in consumer brand trust, which in turn reduces purchase intention (Zhang and He, 2025). At the same time, another line of research has revealed a trust repair mechanism in the face of authenticity deficits among virtual influencers. Studies have found that credibility acts as a “psychological repair mechanism” in virtual endorser marketing: when consumers perceive that a virtual influencer lacks authenticity, they can compensate for this deficit by strengthening their perceived credibility of the information source, thereby achieving persuasive effects (Zhou et al., 2026). The research further reveals the differentiated effects of authenticity in the virtual influencer context, showing that consumers can separate “high influencer authenticity” from “low claim authenticity”. This indicates that the effect of brand trust on consumers in the virtual endorser context is not unidimensional. Building on these theoretical insights, this study further deepens the understanding of the relationship between virtual influencers and brand trust. We extend brand trust from the traditional two-party relationship of “brand consumer” to a triadic relationship of “brand virtual endorser consumer”. Drawing on trust theory, this study reveals how the stability and controllability of virtual idols enhance consumer trust in functional brands and subsequently affect brand attitude. This theoretical extension not only validates the applicability of classic theories in emerging digital endorsement contexts but also provides empirical evidence for future research combining virtual images, digital endorsement, and consumer psychological mechanisms. It demonstrates that brand trust can be established not only through direct brand consumer interaction but also indirectly through virtual endorsers. In summary, by investigating the influence of virtual idol endorsers on consumer brand attitude based on meaning transfer theory and brand trust theory, this study not only extends the application scenarios of the two theories but also reveals that they function as complements rather than substitutes contingent on contexts: for symbolic products, meaning transfer dominates the effect of virtual idol endorsers on consumer brand attitude; for functional products, trust building dominates.

This study further reveals the moderating role of brand type in the influence path of virtual idol endorsers on consumer brand attitude, that is, the boundary conditions of this effect responding to the call by (Thomas and Fowler 2021) for deeper exploration of the differential impact of virtual endorsers across different product categories. Although previous research has examined the role of AI recommendations or virtual agents in consumer choice, the application of virtual idols in endorsement remains a relatively new topic (Kong and Fang, 2024; Longoni and Cian, 2022; Yoon and Lee, 2021). Moreover, prior studies have often treated brands holistically, for example, focusing on luxury or new products. Research on how to balance the characteristics of symbolic and functional products to shape consumer attitudes toward products endorsed by virtual idols remains relatively scarce (Liu et al., 2024; Sheehan, 2020). Brand type is an important perspective for analyzing different psychological processes in consumer purchase decisions (Mishra et al., 2021). Therefore, this study provides a new explanatory mechanism and extends the application domain of brand type, contributing to the understanding of how the interplay between product attributes and virtual endorsers affects consumer brand attitude.

### Managerial implications

Compared with the unpredictable risks of negative events associated with real endorsers, the advantages of virtual idol endorsers, such as stability, controllability, and malleability, can significantly enhance the stability of brand endorsements and the sustainability of brand marketing and promotion activities (Thomas and Fowler, 2021; Yang et al., 2023). Enterprises should fully realize the potential of virtual idol endorsers, and actively explore the application scenarios of virtual idol endorsers in combination with their own brand characteristics and the needs of target audiences, so as to optimize brand marketing strategies and enhance brand competitiveness (Franke et al., 2023).

With virtual idol endorsers as the media and assistance, enterprises can better express their brand culture and product characteristics, convey their own brand image and product value, make consumers feel the brand appeal, and influence consumers' attitude subtly. Therefore, by deconstructing the influence process of virtual idol endorser, this study guides enterprises to optimize their use of virtual idol endorsers, realize the publicity and promotion of brand image, and enhance their profitability *via* the image and influence of virtual idols.

Brands need to tailor their use of virtual endorsers to the characteristics of different types of products. This paper verifies that when consumers purchase functional brand products, brand trust plays a positive role in the process of virtual idol endorsers affecting brand attitude; when buying symbolic brand products, social identity has a significant impact. Therefore, enterprises should pay attention to the psychological needs of consumers so as to improve the brand marketing effect. For functional brands, virtual idol spokespersons should be used to strengthen consumers' brand trust, such as showing the product research and development process, quality assurance measures and so on, so as to improve consumers' trust in the product performance. For symbolic brands, businesses should focus on meeting consumers' needs for social identity, and emphasize the unique values of brands and social status symbols. They can further boost the effect of brand marketing and strengthen consumers' brand loyalty by enhancing consumers' emotional resonance to the brand and optimizing consumption experience.

## Limitations and directions for future research

The brand samples selected in the experimental design are relatively limited. Whether it is electronic products / aviation service products or automobile / sports brands, they are only a part of many brands, and it is difficult to fully cover the diversity in different industries, scales and development stages. This may potentially compromise the generalizability of the findings. In addition, this study divides brand types into functional and symbolic ones, but the classification of brand types in the actual market may be more complicated, such as dominant or emerging brands, innovative or conservative brands, experience products or search products. Future research can further refine the classification of brand types and explore the influence mechanism of virtual idol endorsers under different segmentation types.

The study adopts a predominantly single-method approach. Scenario experiment method is employed mainly, which can control variables and verify hypotheses to some extent, but lacking the supplement of other research methods. It is difficult to fully capture the behavioral and psychological changes of consumers in actual purchase scenarios only by relying on scenario experiments. In the actual consumption decision-making process, consumers may be comprehensively affected by a variety of factors, such as purchase channels, product prices, and purchase convenience, which are difficult to fully simulate in the experiment and may lead to deviations between the research results and the actual situation. With a range of research methods, such as field research and interviews, future research can collect data from multiple perspectives through field observations of consumers' behavioral patterns in authentic purchasing contexts, complemented by in-depth interviews to uncover their psychological mechanisms and decision-making processes, to achieve a more comprehensive and precise understanding of consumer behavior and underlying psychological frameworks.

The variables lack sufficient depth in investigation. This study mainly focuses on the two mediating variables of social identity and brand trust, and they are only partial mediators, not complete ones, and also the moderating effect of brand type is conditional. Beyond the boundary, social identity and brand trust are likely to be covered by other influencing factors and thus fail to play a role. In addition, the formation mechanism of consumer brand attitude may be more complex, involving more potential variables, such as brand attachment, brand awareness, brand loyalty, brand strength and so on. Future research can further expand the variable system and include more mediating or moderating variables to reveal the influencing mechanism of virtual idol endorsers on consumer brand attitude more comprehensively.

## Data Availability

The original contributions presented in the study are included in the article/supplementary material, further inquiries can be directed to the corresponding author.

## Appendix

**Table A1:** Measurement scale.

Variables	Items	Source
Brand attitude	I think this brand is professional.	(Ohanian 1990)
	I think this brand is of high quality.	
	I think this brand is trustworthy.	
	I think this brand is satisfactory	
	I think this brand is attractive	
	I think this brand is likable.	
Social identity	I think the concept conveyed in this brand is the mainstream concept of society.	(Cheek et al. 1994); (Johnson et al. 2004)
	Buying the products of this brand makes me feel that I have become one of the vast majority.	
	The values conveyed by this brand make me feel a strong sense of belonging.	
	Identifying with the brand makes me feel more confident about my attitude toward life.	
	If I live my life like the people in the picture, others will recognize and like me.	
	I think the use of the brand's products or services can be as popular as the people in the picture.	
Brand trust	I think this brand meets my expectations.	(Delgado-Ballester et al. 2003)
	I have great confidence in this brand.	
	I think this brand has never let me down.	
	I think the high satisfaction of this brand is obvious to all.	
	This brand would be honest and sincere in addressing my concerns.	
	I could rely on this brand to solve the problem.	
	This brand would make any effort to satisfy me.	
	This brand would compensate me in some way for the problem with the product.	
Symbolic brand	The products of this brand match my personality.	(Sweeney and Soutar 2001); (Wallace et al. 2014)
	I think that this product is used by people like me.	
	The products of this brand fit my social role.	
	The products of this brand can improve others' impression of me.	
	The products of this brand can make me more confident in social interactions.	
Functional brand	This product has many functions and I'm very satisfied.	(Sweeney and Soutar 2001)
	After using the products of this brand, I am satisfied with its performance.	
	The overall quality of the products of this brand is very good.	
	The products of this brand are very convenient and easy to use.	
	The products of this brand are relatively durable.	
